# Keystone *K*-Strategists Shape Microbial Co-Occurrence Networks in Shale-Gas Wellpad Soils in the Field, Sichuan Basin, China

**DOI:** 10.3390/biology15201836

**Published:** 2026-10-14

**Authors:** Jie Xu, Yi Liu, Jian Yun, Ximing Cheng, Erxiao Wang, Chunling Tang

**Affiliations:** 1 Safety and Environmental Protection Supervision and Testing Center, PetroChina Southwest Oil & Gasfield Company, No. 12, Tianfu North Ave., Wuhou District, Chengdu 610051, China; 2 Sichuan Key Laboratory of Shale Gas Geology Evaluation and Efficient Exploitation, National R&D Center of High-Sulfur Gas Reservoirs, No. 218, Tianyan Rd, Shuangliu District, Chengdu 610213, China; 3 Research Institute of Safety and Environmental Protection Technology, PetroChina, No. 1, Huanghe North St., Changping District, Beijing 102206, China; 4 Faculty of Education, Monash University, Melbourne, VIC 3800, Australia

**Keywords:** shale-gas wellpad soil, 16S rRNA gene sequencing, chronic disturbance, molecular ecological network, keystone species, *K*-strategy

## Abstract

Shale-gas extraction imposes persistent, low-intensity, multi-factor disturbances on terrestrial soil ecosystems, driven by flowback-fluid-derived salinization, drilling-residue deposition, and moderate petroleum hydrocarbon contamination. However, microbial community assembly mechanisms and the corresponding ecological strategies under such combined chronic perturbations remain insufficiently understood. This study employed 16S rRNA gene sequencing, quantitative PCR, and molecular ecological network (MEN) analysis to characterize the soil microbiome within the Changning shale-gas field, Sichuan Basin, China. The results revealed that microbial communities in shale-gas wellpad soils formed highly modular (M = 0.786), non-random “small-world” co-occurrence networks, indicating intrinsically stable microbial systems. Via *Z_i_*-*P_i_* topological classification, six low-abundance (<0.63%) keystone taxa were identified: three module hubs (OTU11572, OTU14908, OTU11976) and three connectors (OTU8845, OTU13035, OTU8962), phylogenetically affiliated with *Acidobacteriota*, *Chloroflexi*, and *Actinobacteriota*. These keystone organisms exhibited typical *K*-strategy traits, including high network connectivity (degree = 21–24), metabolic stability, and dependence on inter-taxa cooperative interactions. Significant positive correlations were detected between these *K*-strategy keystone species and anaerobic functional genes (*nirS*, *nirK*, *dsrAB*) responsible for denitrification and sulfate reduction, highlighting their adaptation to fluctuating redox environments generated by drilling-cutting accumulation. Distance-based redundancy analysis (Db-RDA) further demonstrated that electrical conductivity (representing salinization stress from flowback-water intrusion) and soil organic matter acted as the dominant environmental filters shaping microbial community composition, outweighing the influence of labile inorganic nutrients. Overall, this work provides novel evidence that *K*-selection dominates microbial community assembly under chronic soil disturbance associated with shale-gas extraction. Keystone *K*-strategists sustain modular network stability through the combination of metabolic plasticity and anaerobic respiratory adaptation. These findings expand *r*/*K*-selection theory to anthropogenically disturbed soil habitats and offer theoretical guidance for soil ecological restoration and risk assessment for shale-gas exploiting regions.

## Simple Summary

Soil microbial responses to chronic, low-intensity multi-stress disturbance from shale-gas exploitation at the field sites were investigated in this study. This work distinguishes shale-gas-driven chronic salinization-dominated perturbation from acute high-magnitude petroleum-hydrocarbon pollution, a disturbance type that typically favors fast-growing *r*-strategist degraders. The study combines high-throughput amplicon sequencing, qPCR quantification of functional genes, and MEN-based co-occurrence network analysis to characterize microbial community assembly patterns. Topological *Z_i_*-*P_i_* classification identified several keystone taxa from oligotrophic phyla. Db-RDA and variance-partitioning results confirmed that salinity-related electrical conductivity and soil organic matter, rather than readily available mineral nutrients, are the main abiotic drivers shaping the distribution of keystone taxa. This study further proposes a “disturbance-stability” hypothesis, extends the application of the *r*/*K*-selection framework to soil microbiomes under such persistent multi-stress disturbances, and discusses practical management implications for impacted sites. We also acknowledge some limitations of this study, including the limited sample size and the use of correlation-based network inference, and propose targeted directions for future research.

## 1. Introduction

Shale-gas extraction has emerged as a major component of global energy supply portfolios [1], and China hosts extensive shale-gas reserves, the majority of which are concentrated in the Sichuan Basin. The Changning–Weiyuan National Shale-Gas Demonstration Zone has established an annual production capacity exceeding 14.6 billion cubic meters, with over 2000 wells drilled since 2009 [2,3]. However, shale-gas development introduces complex disturbances to local soil ecosystems, including drilling fluid leakage, oil-based cuttings accumulation, and wastewater runoff during precipitation events. Unlike acute oil spills, these disturbances are characterized by low concentrations, chronic persistence, and multi-source contamination, which exert continuous cumulative pressures on the structure and function of soil microbial communities [1,4]. Understanding the assembly mechanisms and stability maintenance of microbial communities under such persistent disturbances is critical for conducting ecological risk assessments and formulating soil management strategies in shale-gas development regions [5,6].

Soil microorganisms assemble into complex interaction networks via competition, cooperation, and metabolic interdependence [7], and the topological properties of these networks are closely associated with community stability and functional capacity in impacted environments. These disturbances associated with shale-gas wellpads differ fundamentally from acute petroleum spills. Acute spills are pulsed contamination events characterized by high magnitude, driven by toxic hydrocarbons that impose strong chemical filtering and rapid soil-biota mortality, ultimately selecting for stress-tolerant ruderal *r*-strategist taxa [5]. In contrast, wellpad disturbance stems from chronic physical alterations including topsoil removal, soil compaction, and habitat homogenization, in which habitat loss outweighs chemical toxicity. This perturbation imposes sustained habitat filtering rather than short-term toxic shock.

Most existing frameworks of *r*/*K*-selection and community assembly are derived from ecosystems impacted by spill disturbances; empirical evidence linking microbial network topology, keystone taxa dynamics, and *r*/*K*-strategist responses under chronic physical land-use disturbance remains underrepresented [6]. Molecular ecological network (MEN) analysis robustly identifies keynote taxa, which are species that disproportionately structure microbial communities despite their low abundance [8,9]. Based on OUT-level Spearman correlations and node classification via within-module connectivity (*Z_i_*) and among-module connectivity (*P_i_*), MEN categorizes nodes into four types: module hubs (*Z_i_* > 2.5), connectors (*P_i_* > 0.62), network hubs, and peripheral nodes [10,11]. These keystone taxa occupy central, highly connected positions that govern network stability and ecosystem function. Although MEN analysis has been applied to heavy-metal-polluted soils, agroecosystems, and wastewater treatment bioreactors, it has not yet been explored for soils surrounding shale-gas wellpads. This study fills this critical gap by unraveling microbial community responses to persistent physical land modification, refining interpretations of *r*/*K*-selection theory, and extending the predictive capacity of microbial community assembly theory beyond acute-contamination scenarios.

Ecological theory provides robust conceptual frameworks for interpreting the assembly of microbial communities [8,11]. Under *r*/*K* selection theory, r-strategists are favored under fluctuating resource conditions, exhibiting rapid growth rates but low competitive ability. In contrast, *K*-strategists dominate stable habitats through efficient resource utilization and strong interspecific cooperation [10,12]. For soils subjected to chronic physical disturbance such as shale-gas wellpad development, persistent pools of recalcitrant organic compounds may potentially select for *K*-strategy microorganisms. Such organisms commonly feature high metabolic diversity, extracellular enzyme secretion, and cooperative metabolic traits, enabling them to act as functional cores that maintain the stability of the entire community [8]. However, specific criteria for identifying *K*-strategists in MENs, the corresponding topological characteristic of these taxa, and their adaptations to physically disturbed soil environments remain poorly defined.

Given the distinct disturbance regimes between shale-gas wellpad soils and conventional acute petroleum-contaminated sites, this study was initiated to address critical knowledge gaps regarding the application of *r*/*K*-selection theory to microbial community assembly. We characterized soil microbial communities in the Changning shale-gas field, Sichuan Basin, using high-throughput sequencing combined with quantitative PCR (qPCR). We further constructed MEN to identify keystone species and characterize their ecological strategies. In addition, we quantified the abundance of denitrification genes (*nirS*, *nirK*) and sulfate reduction genes (*dsrAB*) to explore potential associations between *K*-strategy taxa and microbial anaerobic adaptation. The specific objectives of this study were: (1) to characterize microbial community structure and network topology in shale-gas wellpad soils; (2) to identify keystone species and determine their ecological strategies; and (3) to elucidate the relationship between *K*-strategy and microbial environmental adaptation. This research aims to reveal assembly mechanisms and stability maintenance strategies of soil microbial communities under chronic anthropogenic disturbance, and to provide theoretical insights for microbial ecology research in disturbed ecosystems.

## 2. Materials and Methods

### 2.1. Study Area and Sample Collection

The study was conducted in the Changning–Weiyuan National Shale Gas Demonstration Zone, which is located in the southern Sichuan Basin (104°44′–105°03′ E, 28°15′–28°48′ N). Representative wellpads with an operation age of more than 2 years were selected, and nine sampling points were established across three shale-gas sites in Gongxian County and Xingwen County (Figure 1). Soil samples at a depth of 0–20 cm were collected using sterile sampling tools, transported to the laboratory in dry ice containers, and pre-treated through a 2 mm mesh sieve, and then stored separately at −80 °C for subsequent DNA extraction and 4 °C for physicochemical property analysis.

### 2.2. Soil Physicochemical Analysis

After calibrating with standard buffer solutions, soil pH was measured using a PHS-3C pH meter (Shanghai instrument scientific instrument Co., Shanghai, China). For the electrical conductivity (EC) assay, the electrode constant was calibrated with a standard KCl solution. Three technical replicates were prepared for each experimental sample, and the final result was calculated as the average of the replicates. Furthermore, soil cations K^+^, Na^+^, Mg^2+^, and Ca^2+^ were quantified via a Super 990F atomic absorption spectrophotometer (AAS, Beijing Puxi General Instrument Co., Beijing, China). Soil anions Cl^−^, SO_4_^2−^, and NO^3−^ were measured using a DX-300 ion chromatographer (Dionex, Sunnyvale, CA, USA) fitted with an AS4A-SC anion chromatography column and a CDM-II conductivity detector, with 1.7/1.8 mol/L sodium carbonate/sodium bicarbonate solution at a flow rate of 2.0 mL/min [13]. Prior to detection, soil organic carbon samples were filtered through 0.22 μm syringe filters (Jinteng, Tianjin, China) and analyzed using a TOC/TN Analyzer (Multi N/C 3100, Analytik, Jena, Germany).

### 2.3. DNA Extraction, Sequencing and Quantitative PCR

Total DNA was extracted using the E.Z.N.A.^®^ Soil DNA Isolation Kit (Omega Bio-tek, Norcross, GA, USA) following the manufacturer’s protocols. DNA quality was assessed via 1% agarose gel electrophoresis. The V3-V4 hypervariable region of the 16S rRNA gene was amplified with primers 338F (5′-ACTCCTACGGGAGGCAGCAG-3′) and 806R (5′-GGACTACHVGGGTWTCTAAT-3′). Paired-end sequencing (2 × 250 bp) was performed on the Illumina NovaSeq PE250 platform (Shanghai Majorbio Bio-pharm Technology Co., Shanghai, China). Raw sequencing reads were processed using QIIME 2: DADA2 for denoising and chimera removal, clustering into ASVs/OTUs at 97% similarity, and taxonomic assignment was performed against the SILVA 138 database on the Majorbio Cloud Platform (www.majorbio.com). Moreover, the abundance of functional genes (*nirS*, *nirK*, *dsrAB*) was quantified using an ABI 7500 Real-time PCR System (Applied Biosystems, Waltham, MA, USA). Primer sets cd3aF/R3cd, F1aCu/R3Cu and dsrA-F/dsrB-R were used for the amplification of *nirS, nirK* and *dsrAB*, respectively. The 20-μL qPCR reaction mixture consisted of 10.0 μL of 2 × SYBR Green qPCR Mix, 0.8 μL of each primer (10 μM), 1.0–2.0 μL of template DNA, and nuclease-free water. The amplification procedure included an initial denaturation step at 95 °C for 5 min, followed by 40 cycles of 95 °C for 30 s, 57 °C for 45 s, 72 °C for 45 s for *nirS* and *nirK*; the annealing temperature was set at 58 °C for *dsrAB*. Melting-curve analysis was performed to confirm amplification specificity. Standard curves were constructed using serially diluted plasmids inserted with target gene fragments. Negative-template controls were included in each amplification run.

### 2.4. Co-Occurrence Network Analysis

To decipher the symbiotic association patterns of soil microorganisms and the composition of petroleum hydrocarbon-related communities in soils at shale-gas well sites, systematic MENs (pMENs) were constructed and analyzed using a random matrix theory (RMT)-driven framework [11]. The MEN analysis platform (http://ieg4.rccc.ou.edu/mena/, accessed on 1 July 2026) was used to implement RMT-based network construction for soil microbial OUT datasets obtained from shale-gas field soils [14]. Briefly, co-occurrence association networks were built on the basis of Spearman’s rank correlation coefficients (|ρ| > 0.7, *p* < 0.01) among microbial OTUs. The network outputs were imported into Cytoscape software (v3.2.1) for visualization of interaction architectures and calculation of core topological parameters, including node degree, clustering coefficient, modularity index, and path length. In each network graph, an individual node represents a microbial OUT that potentially performs distinct ecological functions in the contaminated soils, while edges indicate statistically significant pairwise correlations between microbial taxa. Multiple network-level metrics were further extracted to characterize the topological properties of microbial networks in shale-gas wellpad soils: total node number, total edge number, node degree, betweenness centrality, overall connectivity, average clustering coefficient, average geodesic distance, and modularity. In the microbial symbiotic network, keystone microbial taxa are generally defined as taxa with high node degrees and low betweenness centrality values [10]. Module-based node classification was performed by calculating two key indices: within-module connectivity (*Z_i_*) and among-module connectivity (*P_i_*). Nodes were accordingly partitioned into four ecological categories: module hubs (*Z_i_* > 2.5), connectors (*P_i_* > 0.62), network hubs (*Z_i_* > 2.5 together with *P_i_* > 0.62), and peripheral nodes.

### 2.5. Statistical Analysis

To investigate the relationships between soil microbial community assembly and soil physicochemical properties under shale-gas-derived petroleum-hydrocarbon contamination, the Mantel test and non-metric multidimensional scaling (NMDS) were conducted using SPSS (v25.0). Microbial community matrices were preprocessed before analyses to fit the polluted soil datasets collected from shale-gas wellpads. We further applied Spearman’s rank correlation analysis to quantify pairwise associations between individual soil environmental variables and microbial community profiles and to identify key physicochemical drivers that shape microbial community structures in contaminated soils from shale-gas exploitation sites.

## 3. Results and Discussions

### 3.1. Microbial Community Structure, Diversity and Taxonomic Characteristics

High-throughput 16S rRNA sequencing yielded a total of 1,247,583 qualified high-quality bacterial sequences, which were clustered into 12,456 OTUs (at 97% similarity threshold). Beta-diversity patterns of bacterial communities across different sampling zones were visualized using NMDS based on Bray–Curtis dissimilarity matrices (Figure 2). The NMDS ordination revealed clear spatial separation of microbial assemblages among sampling points: the first axis (NMDS1) explained 36.4% of the total community variation, while the second axis (NMDS2) accounted for 21.7% of the variation. Specifically, NMDS1 predominantly reflected geographical spatial gradients across sampling sites, whereas NMDS2 was strongly correlated with key soil physicochemical properties. This indicates that both spatial location and edaphic variables jointly structured the bacterial community composition among sampling groups (ANOSIM: R^2^ = 0.326, *p* = 0.0399). The moderate ANOSIM R value suggests a statistically significant but subtle difference in community composition, which is a typical characteristic of microbial communities shaped by continuous environmental gradients rather than discrete habitat differentiation [15].

Alpha-diversity analysis detected significant differences in community diversity and dominance between the disturbed zones and the buffer zones. The Shannon diversity index, a comprehensive metric capturing both species richness and evenness, was significantly lower in the core wellpad disturbance zone (5.23 ± 0.41) than in the undisturbed buffer zones (6.08 ± 0.35) (*p* < 0.05). By contrast, the Simpson dominance index followed the opposite trend, with higher community dominance observed in the core disturbance zone. These divergent alpha-diversity patterns demonstrate that long-term environmental disturbance associated with wellpad operation significantly reduces the biodiversity and species evenness of microbial communities, while enhancing the competitive dominance of disturbance-tolerant taxa. Such community shifts indicate that chronic anthropogenic disturbance filters out sensitive microbial species, simplifies community structure, facilitates the formation of low-diversity, yet relatively stable, microbial assemblages with strong environmental adaptability [16].

Taxonomic composition analysis at the phylum level revealed distinct community structural specificity in wellpad-affected soils (Figure 2a). The dominant bacterial phyla across all samples were *Chloroflexi* (26.0%), *Acidobacteriota* (23.3%), *Proteobacteria* (14.2%), and *Actinobacteriota* (11.7%), which collectively accounted for 75.2% of the total abundance. Notably, *Chloroflexi* and *Acidobacteriota* were significantly enriched in the core disturbed soils (*p* < 0.01) compared with the buffer zones. Consistent with previous soil microbial studies, these two phyla are typical oligotrophic *K*-strategists, characterized by slow growth rates, low metabolic turnover, strong environmental tolerance, and efficient utilization of recalcitrant organic matter in resource-limited habitats [17,18]. Their significant enrichment under long-term disturbance further confirms that persistent environmental stress eliminates copiotrophic *r*-strategists (which feature rapid growth and weak stress resistance), and selectively retains stable, disturbance-resistant *K*-strategists. This process drives the reassembly of soil microbial communities toward an oligotrophic and stress-tolerant assembly pattern.

At the genus level, *Sphingomonas* (4.8%), *Mycobacterium* (3.2%), and *Alkanindiges* (2.1%) are the core indicator taxa of soil microbial communities in wellpad areas, and can clearly distinguish disturbed wellpad soils from undisturbed buffer soils. *Sphingomonas* shows a significant positive correlation with electrical conductivity (*r* = 0.67, *p* < 0.05), indicating a strong adaptive capacity to saline-alkaline conditions [19]. As a typical stress-resistant bacterial genus often detected in anthropogenically disturbed soils, *Sphingomonas* has physiological advantages including osmotic adjustment, high salt tolerance, and the efficient degradation of complex organic pollutants, enabling it to thrive in nutrient-poor degraded saline-alkaline soils [20]. Its enrichment in high-conductivity wellpad soils further confirms the deterministic environmental filtering effect of long-term wellpad disturbance, in which saline-alkali stress acts as a key driver that selects and retains salt-tolerant microbial taxa [15]. In contrast to *Sphingomonas*, *Mycobacterium* and *Alkanindiges* possess unique ecological functions adapted to disturbed soil environments. *Mycobacterium* is an oligotrophic *K*-strategic taxon characterized by slow growth, strong tolerance to nutrient deficiency and environmental stress, which is consistent with the dominant survival strategy of core phyla enriched in wellpad soils. Meanwhile, the genus *Alkanindiges* is known for its ability to utilize hydrocarbon pollutants, allowing it to colonize soils affected by long-term industrial disturbance and degrade residual organic contaminants in wellpad soils [19]. Overall, the co-enrichment of these three indicator genera reflects the dual stress characteristics of wellpad soils: saline-alkali stress and chronic industrial disturbance. Compared with copiotroph-dominated communities in buffer soils, the simplified genus-level community structure in core wellpad zones confirms that persistent anthropogenic disturbance reshapes microbial community assembly: it eliminates disturbance-sensitive *r*-strategic microbes and forms a stress-resistant, oligotrophic microbial consortium dominated by tolerant indicator taxa, which maintains the functional stability of soil microbial ecosystems under long-term environmental pressure [16].

As shown in Figure 3, Welch’s *t*-test identified four OTUs with significant differences in relative abundance (*p* < 0.05): OTU1204 (*Pseudomonadaceae*), OTU120 (*Sulfobacillus*), OTU322 (*Acidibacter*), and OTU201 (*Salinicola*). These differential taxa are involved in sulfur metabolism and saline tolerance, which precisely reflects the dual environmental selection pressures of long-term industrial disturbance and saline-alkali stress in wellpad ecosystems [21]. Consistent with the genus-level indicator profiles observed in this study, the enrichment of salt-tolerant and sulfur-metabolizing microbial taxa further validates that persistent wellpad disturbance induces deterministic microbial community filtering. However, these findings demonstrate that merely characterizing microbial community diversity and taxonomic distribution is insufficient to comprehensively interpret microbial ecological functions and pollutant biodegradation potential in disturbed wellpad soils; core functional populations and key functional genes serve as the pivotal drivers regulating microbially mediated pollutant degradation and soil biochemical cycling [19]. As in other studies, long-term wellpad disturbance causes sustained low-intensity environmental stress, which preferentially enriches oligotrophic, stress-tolerant K-strategic microbes with stable metabolic functions and strong environmental adaptability, rather than fast-growing copiotrophic taxa [7]. Shale gas-disturbed soils consistently show reduced microbial diversity and enriched salt- and pollution-resistant functional bacteria, which is highly consistent with the community simplification and stress-adaptive succession observed in this study. In contrast, soils affected by acute crude oil leakage have distinct microbial response patterns. Acute oil pollution causes drastic short-term environmental perturbation, rapidly triggering the explosive proliferation of *r*-strategic hydrocarbon-degrading microbes such as *Pseudomonas* and *Bacillus* [22]. This leads to transiently elevated community abundance but severely reduced community stability. Such acute disturbance typically induces opportunistic microbial succession dominated by pollutant-degrading specialists, with weak selective pressure on saline-tolerant and sulfur-metabolizing taxa.

### 3.2. Co-Occurrence Network Construction and Keystone Species Identification

As shown in Figure 4, the MEN was constructed based on Spearman analysis, containing 892 nodes and 10,744 edges (|ρ| > 0.7, *p* < 0.01). Key network topological parameters were calculated as follows: average degree = 24.0, average path length = 5.29, clustering coefficient = 0.44, and modularity (M) = 0.786. Compared with randomly generated networks of identical size (M = 0.27), the empirical network exhibited significantly higher modularity and non-random co-occurrence patterns (*p* < 0.001), which is consistent with typical “small-world” topological characteristics. Such network signatures reflect strong species cohesion and high functional redundancy within wellpad soil microbiomes under long-term industrial disturbance [23]. The node-degree distribution fitted well to a power-law model (R^2^ = 0.77), demonstrating a distinct “core-periphery” organizational structure, in which a small set of core microbial taxa serves as network hubs and maintains connections with a large number of peripheral subordinate taxa.

Moreover, this highly modular small-world network architecture aligns with observations of soils disturbed by long-term shale-gas exploitation: persistent low-intensity anthropogenic stress drives microbial communities to assemble into compartmentalized, resilient networks dominated by stress-tolerant oligotrophic taxa. Chronic industrial filtering strengthens inter-taxon cooperation within modules and enhances functional compensation, even as overall community alpha-diversity declines [7]. By contrast, soils affected by crude-oil spills show divergent network characteristics: abrupt pulse-type pollution triggers the rapid proliferation of *r*-strategic hydrocarbon-degrading specialists, accompanied by reduced modularity, fewer stable core hubs, and more volatile interspecies associations [22]. In these scenarios, network interactions are mainly driven by opportunistic bloom-forming taxa, leading to transient high network connectivity but low long-term ecological stability. The core-periphery structure observed in this work further supports the earlier inference that chronic wellpad disturbance selects for stable *K*-strategist consortia, where core taxa maintain network integrity, and peripheral taxa provide functional buffering against fluctuating environmental conditions.

Furthermore, six keystone species were identified via *Z_i_*-*P_i_* classification (Table 1): three module hubs (OTU11572, OTU14908, OTU11976) and three connectors (OTU8845, OTU13035, OTU8962). The identified module hubs were primarily affiliated with *Acidobacteriota* and *Chloroflexi*, exhibiting high within-module connectivity (degree = 21–24) and functioning as core “resource processors” that maintain stable material and energy cycling within individual functional modules. In contrast, connector taxa spanned multiple network modules and acted as critical bridges enabling cross-module material exchange and functional complementation, effectively sustaining the overall integrity and connectivity of the entire microbial co-occurrence network. Notably, all identified keystone species presented extremely low relative abundances (<0.63%, mean = 0.18%), which provides strong support for the classic “low abundance–high impact” ecological paradigm. This phenomenon is consistent with previous findings in long-term disturbed agricultural and industrial soils, confirming that rare, rather than dominant, microbial taxa act as pivotal regulators of ecosystem stability and multifunctionality under persistent environmental stress [19,24,25].

Ecological strategy analysis further verified that these keystone species possessed typical *K*-strategy characteristics adapted to chronic wellpad disturbance, which fundamentally shaped the high-resilience network structure observed in this study. First, these core taxa exhibited metabolic stability: the *Acidobacteriota* OTU11572 (*Granulicella*) is a typical slow-growing oligotrophic bacterium harboring complete metabolic pathways for recalcitrant carbon degradation, enabling it to survive in nutrient-poor disturbed soils. Second, they relied on efficient interspecies cooperation to resist environmental pressure: *Chloroflexi* OTU14908 displayed a strong positive correlation with the nitrogen-fixing Proteobacteria OTU14948 (*Beijerinckiaceae*) (*ρ* = 0.82), suggesting that cross-taxon mutualism provides sufficient nitrogen resources to support the growth and metabolic activity of oligotrophic keystone taxa [26]. Third, these core microbes exhibited superior environmental tolerance: *Actinobacteriota* OTU11831 (*Acidothermus*) encodes abundant stress-responsive proteins, conferring strong adapatability to saline-alkali stress and long-term industrial perturbation in wellpad soils.

These *K*-strategic traits of keystone species are distinctly different from the ecological strategies of *r*-strategic opportunists (e.g., *Pseudomonas* within Gammaproteobacteria), which are often enriched in soils under acute oil contamination. Opportunistic *r*-strategists can proliferate under short-term nutrient enrichment but exhibit low network connectivity and unstable interspecific interactions; thus, they cannot maintain long-term community stability [27]. Consistent with observations in shale-gas-disturbed ecosystems, chronic industrial filtering selectively retains low-abundance, stress-resistant *K*-strategic keystone taxa, which can reconstruct microbial co-occurrence networks characterized by high modularity and strong functional redundancy. This process effectively compensates for the loss of community diversity caused by long-term disturbance, sustains the functions of soil biochemical cycling, and ultimately drives the formation of simplified yet ecologically stable microbial communities in industrially disturbed soils. This finding further clarifies the unique deterministic community assembly pattern of wellpad soils and differentiates chronic industrial disturbance from acute pollution stress.

### 3.3. Association Between K-Strategy Species and Anaerobic Adaptation

Shale gas wellpad soils frequently develop localized anaerobic microenvironments due to drilling fluid residues and cuttings coverage [28], and microbial anaerobic adaptation is crucial for sustaining community structure and ecological function under long-term industrial perturbation. To further characterize the functional adaptation of microbial communities in chronically disturbed wellpad soils and clarify their consistency with typical shale-gas disturbance scenarios, we quantitatively determined the abundances of key anaerobic functional genes, including denitrification genes (*nirS*, *nirK*) and sulfate reduction genes (*dsrAB*), which encode core enzymes mediating anaerobic respiratory processes (Table 2). The qPCR quantification results revealed that the copy numbers of *nirS, nirK* and *dsrAB* ranged from 2.1 × 10^6^ to 8.7 × 10^7^ copies/g dry soil, 1.5 × 10^6^ to 5.3 × 10^7^ copies/g, and 8.2 × 10^5^ to 3.6 × 10^7^ copies/g, respectively. All functional gene abundances exhibited significant spatial heterogeneity across different sampling zones (*p* < 0.05), which was tightly associated with the variable accumulation of drilling cuttings and heterogeneous physicochemical microenvironments among wellpad sites [29].

Notably, significant correlations were detected between keystone microbial taxa and functional gene abundances, which uncovered the core metabolic mechanisms that support network stability under chronic disturbance. The module hub OTU11572 (classified under *Acidobacteriota*) was positively correlated with *nirS* abundance based on Mantel test analysis (r = 0.43, *p* = 0.02). Members of *Acidobacteriota* are widely recognized as facultative anaerobes capable of performing denitrification under oxygen-limited conditions, enabling them to dominate anaerobic microhabitats in industrially disturbed soils [30]. Meanwhile, the connector OTU8845 (classified under *Chloroflexi*) showed a strong positive association with *dsrAB* abundance (*ρ* = 0.65), and multiple *Chloroflexi* lineages possess complete sulfate reduction metabolic pathways, serving as key contributors to biogeochemical sulfur cycling in anoxic soils [31]. These robust associations between keystone taxa and functional genes demonstrate that *K*-strategy core taxa adapt to frequent redox fluctuations in wellpad soils via flexible anaerobic respiratory metabolism. The integration of anaerobic functional pathways further enhances their metabolic stability and environmental adaptability, which is essential for maintaining the structural integrity and functional coordination of microbial co-occurrence networks under persistent industrial stress [19].

From an ecological perspective, the widespread anaerobic adaptation of core microbial communities is highly consistent with the survival strategy of *K*-strategists in chronically disturbed environments, and this mechanism is broadly applicable to both shale-gas and oil wellpad disturbed ecosystems. Unlike acute crude oil pollution, which triggers the explosive growth of r-strategic degraders that rely on transient nutrient pulses, long-term wellpad construction and shale-gas exploitation generate relatively stable but oxygen-depleted soil microenvironments, where the rapid proliferation of *r*-strategists no longer holds an ecological advantage. Instead, *K*-strategic microbes gain competitive superiority through efficient energy utilization, strong metabolic plasticity, and stable community cooperation [19]. Although denitrification and sulfate reduction yield less energy than aerobic respiration, these anaerobic pathways can proceed continuously under anoxic or microaerobic conditions, providing persistent and stable energy sources for oligotrophic *K*-strategists to maintain slow but steady growth [32]. This “stability-oriented adaptive strategy” precisely matches the chronic low-intensity disturbance characteristics of oil and shale-gas wellpad soils. Continuous environmental filtering eliminates unstable opportunistic taxa, selectively enriches anaerobic-tolerant *K*-strategic keystone species, and ultimately constructs low-diversity but highly stable, and functionally redundant microbial networks that can resist long-term industrial disturbance [33].

### 3.4. Environmental Drivers of Community and Network Patterns

As illustrated in Figure 5 and Table 3, distance-based redundancy analysis (db-RDA) identified electrical conductivity (explaining 22.4% variance explained), Ca^2+^ (18.7%), Mg^2+^ (16.6%), and NO_3_^−^ (15.3%) as the primary drivers shaping microbial community structure. Further variance partitioning from Table 3 revealed divergent explanatory capacities of soil ions for shared and unique bacterial assemblages: Ca^2+^ exerted the strongest explanatory power (31.5%, *p* = 0.004) for shared bacterial communities across sampling sites, while Mg^2+^ predominantly explained the variation in site-specific unique bacterial communities (20.4%, *p* = 0.044). The Spearman correlation heatmap presented in Figure 5 further visualizes distinct taxon–ion associations: multiple core genera, including *Sphingomonas*, *Pseudomonas*, *Bacillus*, and *Gaiella*, exhibited significant correlations with soil base cations (Ca^2+^, Mg^2+^) and salinity-related variables (EC, K^+^, Na^+^), whereas nitrate, sulfate and carbonate displayed relatively limited regulatory effects on most microbial taxa. Ionic strength, as reflected by EC, was positively correlated with the abundance of *K*-strategy microbial species (r = 0.58, *p* < 0.05), indicating that salinization stress induced by flowback-water intrusion during shale-gas exploitation imposes strong directional selection that favors halotolerant *K*-strategists [34]. As critical modulators of microbial cellular signaling and enzymatic activity, Ca^2+^ and Mg^2+^ can reshape microbial physiological performance and interspecies symbiotic interactions under such polluted soils. Consistent with previous investigations of shale-gas disturbed soils, base cations and secondary salinization have been repeatedly reported as dominant abiotic filters governing microbial community assembly, outperforming conventional nutrient indicators in structuring bacterial assemblages at wellpad sites [35]. Comparable patterns have also been documented in petroleum-hydrocarbon-polluted soils, where salinity and alkaline-earth cations, rather than inorganic nitrogen nutrients, are the primary determinants of the distribution of hydrocarbon-degrading microbiota [36].

The distribution of keystone species showed a significant positive correlation with soil organic matter content (r = 0.55), but only weak correlations with labile bioavailable nutrients (NH_4_^+^, NO_3_^−^). This observation further validates the *K*-strategy trait of these keystone taxa: they preferentially adapt to environments with stable, low-quality organic carbon pools rather than relying on pulsed inputs of readily bioavailable nutrients [19]. This finding provides novel insights into microbial community assembly in disturbed soils. For shale-gas wellpad habitats, which are characterized as “low-nutrient, high-disturbance” ecosystems, the stability of microbial assemblages is maintained via metabolic cooperation among *K*-strategist populations, instead of the rapid boom-and-bust turnover of *r*-strategist microbes [34]. In contrast, many petroleum-polluted sites receiving fresh hydrocarbon inputs are frequently dominated by fast-growing *r*-type degraders that respond rapidly to carbon pulses. This divergence indicates that shale-gas wellpad soils experience persistent salinity–ion disturbance superimposed on relatively moderate hydrocarbon contamination, which selectively enriches stress-tolerant strategists [36].

A high modularity value (M = 0.786) of the MENs demonstrates that pronounced environmental heterogeneity across wellpad soils drives microbial niche differentiation. Individual network modules likely correspond to distinct functional guilds responsible for carbon degradation, nitrogen transformation, and sulfur cycling, respectively. Keystone taxa act as module hubs and connectors that mediate cross-module material and metabolic fluxes. This “module core” topological architecture confers strong ecological resistance on the entire microbial community: a disturbance damaging a single module will not trigger whole-system functional failure, as the remaining modules can sustain biogeochemical processes coordinated by keystone organisms [37]. Similar co-occurrence networks have been reported for shale-gas-impacted soils; nevertheless, most earlier studies merely described network topology without discriminating the ecological strategists (*K*-/*r*-strategy) of module-associated keystone species [38]. Unlike previous studies, this study explicitly links ion-mediated environmental filtering, the keystone functional traits of *K*-strategists, and modular network structure, providing an integrated mechanistic explanation for community stability under combined salinity-hydrocarbon disturbance at shale-gas sites.

As visualized in Figure 6, an integrated correlation network was constructed to uncover the distinct ecological strategies of OTUs spanning three phyla: *Acidobacteriota*, *Chloroflexi*, and *Proteobacteria*. In particular, Proteobacterial OTU11962 and OTU14948 showed strong positive correlations with EC, *nirS* gene abundance, and major base cations (Ca^2+^, Mg^2+^, K^+^). This pattern is consistent with the well-documented halotolerant, metabolically versatile characteristics of this phylum in saline-impacted soils. In contrast, OTU12230_Proteobacteria was negatively correlated with phosphorus and pH, confirming the presence of intra-phylum niche partitioning. Notably, *Acidobacteriota OTU11572* showed robust positive linkages to EC and base cations, challenging the conventional view of this oligotrophic phylum as strictly salinity-sensitive. Meanwhile, *Chloroflexi OTUs* displayed stress-tolerant traits with variable associations with denitrification potential. Our findings are broadly consistent with a previous shale-gas soil study, which reported that base cations and salinity were the main abiotic filters shaping the distribution of keystone taxa, and exerted a stronger effect than nutrient availability alone [16].

Unlike petroleum-hydrocarbon-polluted soils, where labile hydrocarbon carbon inputs select for fast-growing *r*-strategist degraders as keystone taxa, keystone organisms at this shale-gas site are stress-tolerant *K*-strategists shaped primarily by persistent ionic disturbance rather than carbon substrate pulses. These geochemical signatures—elevated EC, sulfate, chloride, and divalent cations—closely match the chemical profile of drilling flowback fluids from shale-gas extraction, where Proteobacteria typically dominate community succession under saline stress. Similarly, in petroleum hydrocarbon-contaminated soils, *Proteobacteria* enrichment is well-documented alongside declines in *Acidobacteriota* and *Chloroflexi*, a pattern driven by hydrocarbon-degrading capacities of genera such as *Pseudomonas* and *Marinobacter*. The co-occurrence of *Proteobacteria* and *nirS*-type denitrifiers in the present study aligns with findings from both shale-impacted and petroleum-polluted systems, where nitrate reduction becomes coupled to anaerobic hydrocarbon metabolism under elevated salinity. Collectively, these patterns indicate that the studied soil has undergone substantial anthropogenic disturbance, with salinity and ionic composition serving as the primary forces structuring the microbial community. The differential responses observed across phyla underscore environment-specific filtering: *Proteobacteria* function as haloadapted copiotrophs, *Chloroflexi* maintain metabolic flexibility under stress, and certain *Acidobacteriota* lineages exhibit unexpected persistence in moderate saline-alkali conditions. While the presence of *Acidobacteriota* in stressed shale-gas soils has been widely reported at the phylum level, the present analysis further pinpoints a specific Acidobacteriota OTU that is closely linked to keystone taxa, advancing beyond general phylum-level descriptive observations [30]. These findings emphasize that microbial reassembly in industrially impacted soils is governed not solely by organic contaminant loads but by the integrated effects of salinity stress, nutrient stoichiometry, and alternative electron acceptor availability.

### 3.5. Theoretical Implications and Management Insights

This study extends *r*/*K* selection theory to anthropogenically disturbed terrestrial ecosystems and proposes a “disturbance-stability” hypothesis for microbial communities in shale-gas wellpad soils. Driven by flowback fluid intrusion and petroleum-hydrocarbon contamination, these sites undergo chronic, low-intensity chemical stress, where persistent ionic salinization acts as a strong environmental filter. This filter favors stable, low-quality organic resources over pulses of labile nutrients, leading to the assembly of microbial co-occurrence networks dominated by *K*-strategy taxa [19]. In contrast to acute petroleum pollution such as oil spills, which selects for fast-growing *r*-strategist hydrocarbon degraders, *K*-strategy-dominated communities in shale-gas wellpad soils confer greater functional stability and resistance to recurring ionic stress. However, these communities exhibit slower metabolic recovery and weaker pollutant removal performance following sudden carbon inputs. This key ecological contrast has important practical implications for in situ soil restoration and risk management. For chronically disturbed wellpad soils, preserving native soil organic matter pools and physicochemical integrity outperforms exogenous labile nutrient addition in maintaining microbial populations and biogeochemical processes [39]. Unregulated labile-nutrient amendments may instead favor opportunistic *r*-strategists, which can disassemble the original modular *K*-strategy network and erode long-term community resilience under sustained salinization.

Notably, several important caveats still limit the generalizability of our current interpretations. First, in the absence of long-term time-series data, we are unable to confirm whether *K*-strategy-dominated communities represent disturbance-driven shifts or pre-existing features of undisturbed local soils. Second, MENs are constructed based on correlation statistics, which cannot distinguish direct biological interactions from indirect covariations caused by shared environmental drivers. We therefore suggest that follow-up studies utilizing metagenomic binning, metabolic reconstruction and targeted microbial cultivation would be valuable to verify the metabolic cooperation among *K*-strategy keystone taxa. Third, our current observations were only obtained from three wellpad sites. Broader sampling across diverse geological conditions, pollution legacies and land-use contexts will help examine the applicability of the “disturbance-stability” hypothesis. We also propose that future research combining stable-isotope probing (SIP) with high-resolution omics can help trace the carbon-source preferences of *K*-strategy species. This approach will further clarify how these taxa stabilize communities under combined salinity–hydrocarbon co-contamination, and provide useful insights for developing tailored remediation for shale-gas-impacted soils.

## 4. Conclusions

This work presents an insightful investigation into soil microbial communities across a gradient of disturbance levels within the Changning shale-gas field in the Sichuan Basin. Our study specifically disentangles microbial co-occurrence network patterns and *K*-strategy-driven community assembly under the influence of long-term multi-factor disturbance from wellpad operations. Here, four key findings are highlighted: (1) Wellpad soils form highly modular (M = 0.786) small-world microbial networks, which indicates that chronic low-intensity disturbance can foster stable, functionally redundant microbial communities, supported by intensive interspecies interactions. (2) Six low-abundance (<0.63%) yet high-connectivity keystone taxa that belong to *Acidobacteriota*, *Chloroflexi*, and *Actinobacteriota* (e.g., OTU11572, OTU14908, OTU11976) are identified as *K*-strategists, characterized by metabolic stability, cooperative intertaxon relationships, and anaerobic adaptive capacity. (3) These *K*-strategists show significant positive associations with anaerobic functional genes (*nirS*, *nirK*, *dsrAB*), which reveals that denitrification and sulfate reduction metabolisms support their adaptation to fluctuating soil redox conditions, and help maintain the overall functional integrity of microbial communities under persistent disturbance. (4) EC and soil organic matter are confirmed to be the primary environmental filters that shape the spatial distribution of *K*-strategists; jointly, ionic strength stress and carbon-source availability drive the selection of stress-tolerant, tightly connected microbial association networks.

From an applied perspective, our findings provide valuable actionable guidance for the ecological management of shale-gas wellpads. First, for heavily disturbed core zones, we suggest that the conservation of indigenous *K*-strategist keystone taxa should be prioritized during ecological restoration, instead of relying solely on the introduction of exogenous microbial inoculants. Second, we found that increasing soil organic matter content and mitigating excessive ionic stress (which is characterized by high EC) are effective engineering measures that support the persistence of keystone *K*-strategists and maintain the stability of microbial co-occurrence networks. Third, we recommend that ecological risk assessments for shale-gas development should expand beyond the exclusive focus on acute petroleum hydrocarbon spills: the chronic combined disturbance effects on keystone microbial taxa and their associated anaerobic biogeochemical processes should be routinely incorporated into risk evaluation frameworks. Overall, this study advances the mechanistic understanding of microbial *r*/*K*-selection under chronic anthropogenic soil disturbance and provides reliable microbial-based decision support for the in situ restoration of shale-gas-impacted soils.

## Figures and Tables

**Figure 1:**
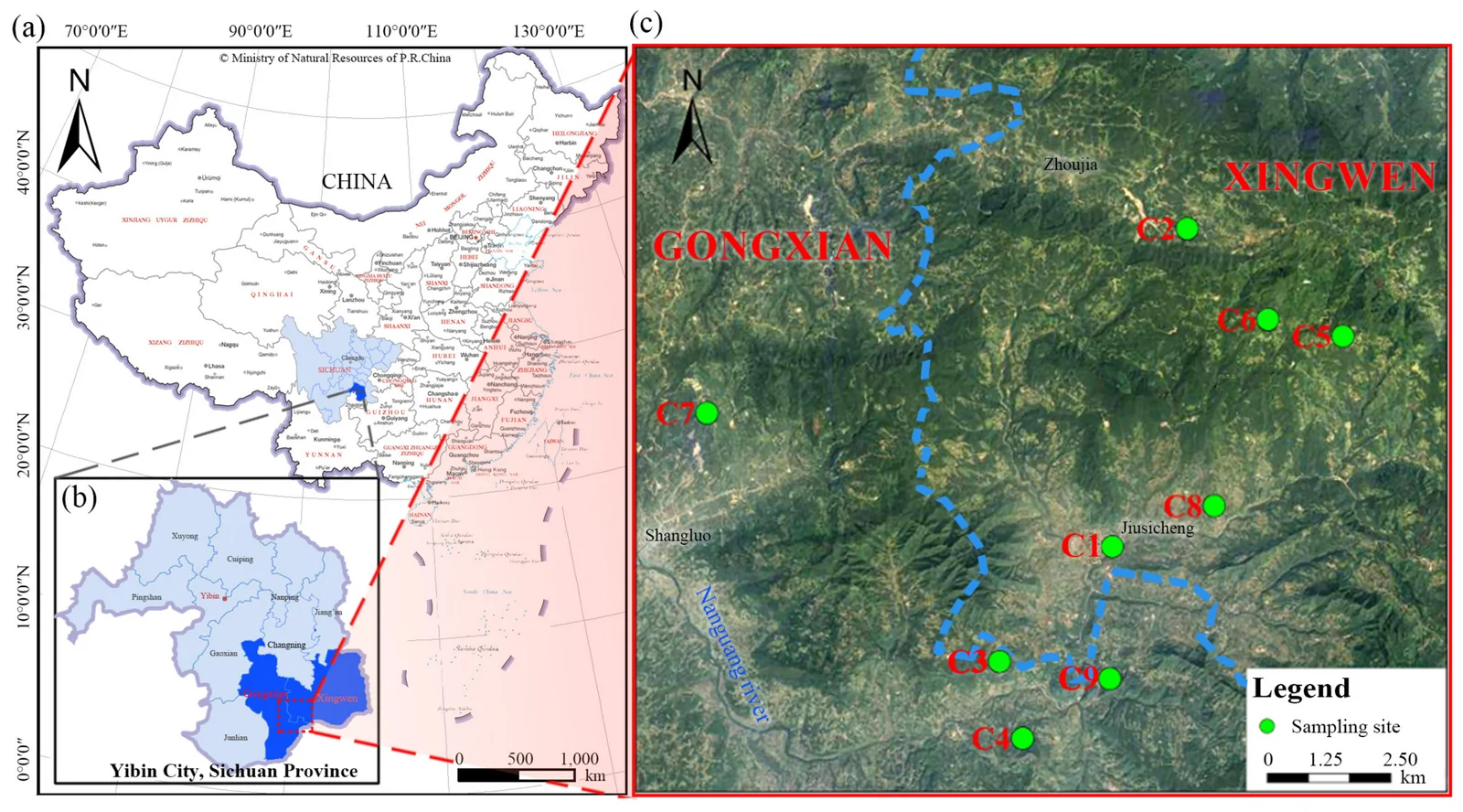
Overview of the study area. (**a**) The location of Sichuan Province in China; (**b**) The location of Gongxian County and Xingwen County in Yibin City, Sichuan Province; (**c**) Topographical characteristics of the sampling points. The study area includes 9 sampling points at shale-gas wellpads across the two counties.

**Figure 2:**
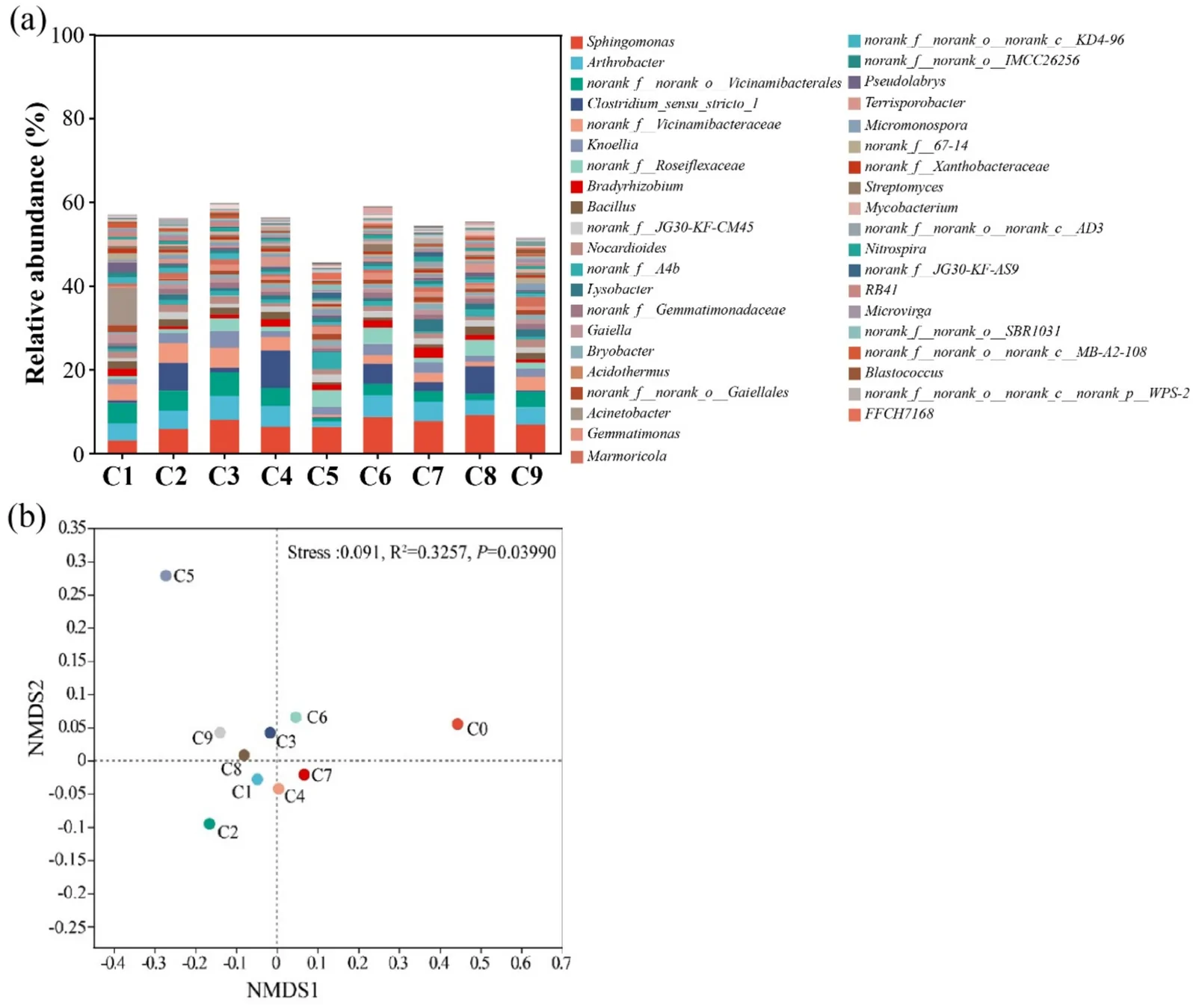
Relative abundance of soil bacterial communities in the study areas at genus level (**a**), and the analysis of bacterial structure conducted via non-metric multidimensional scaling (NMDS) method (**b**).

**Figure 3:**
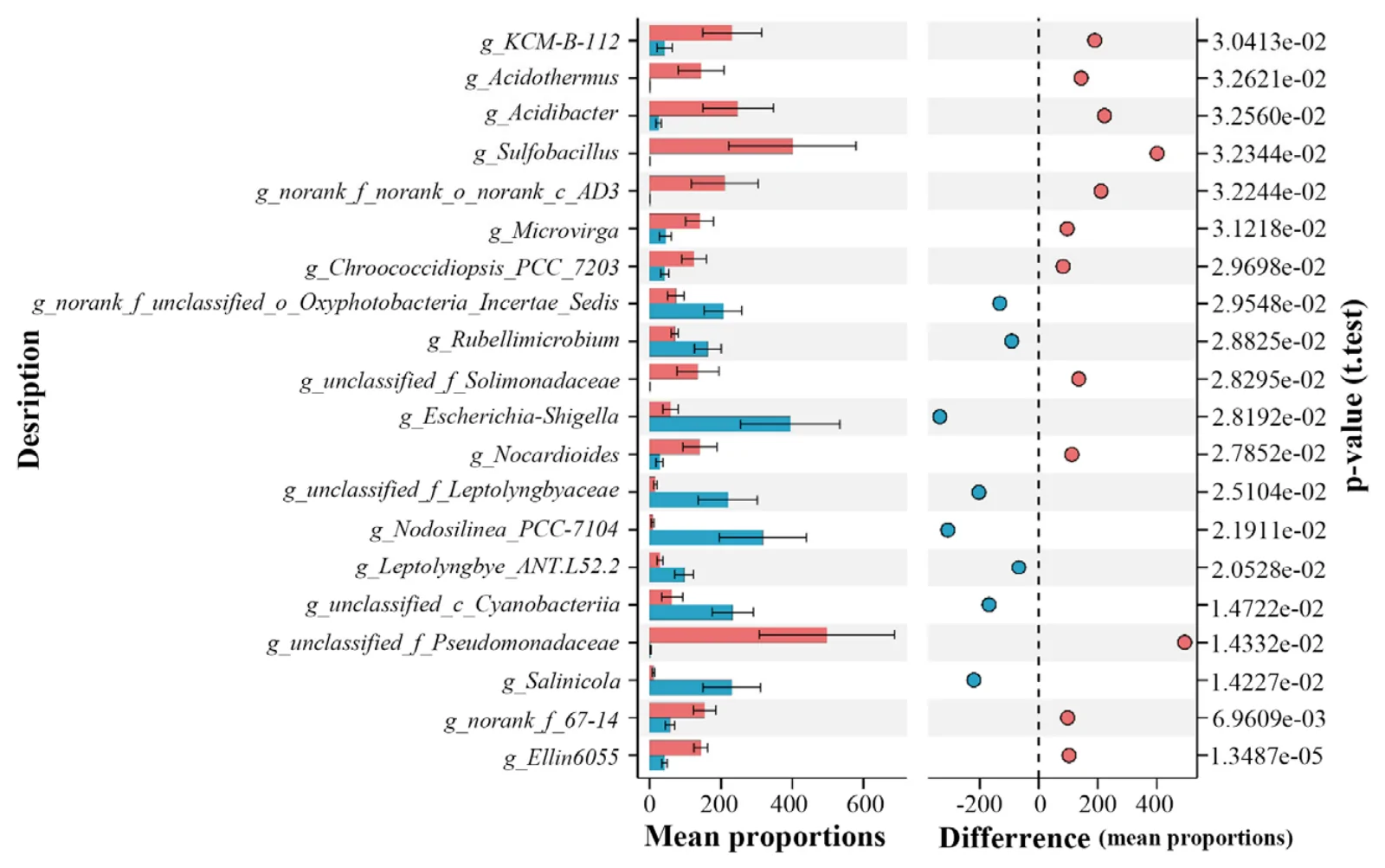
Significance test for intergroup differences among soil microbial communities (Red and blue represent the microbial community and diversity of soil samples, with significant differences, *p* < 0.05; extremely significant differences, *p* < 0.01).

**Figure 4:**
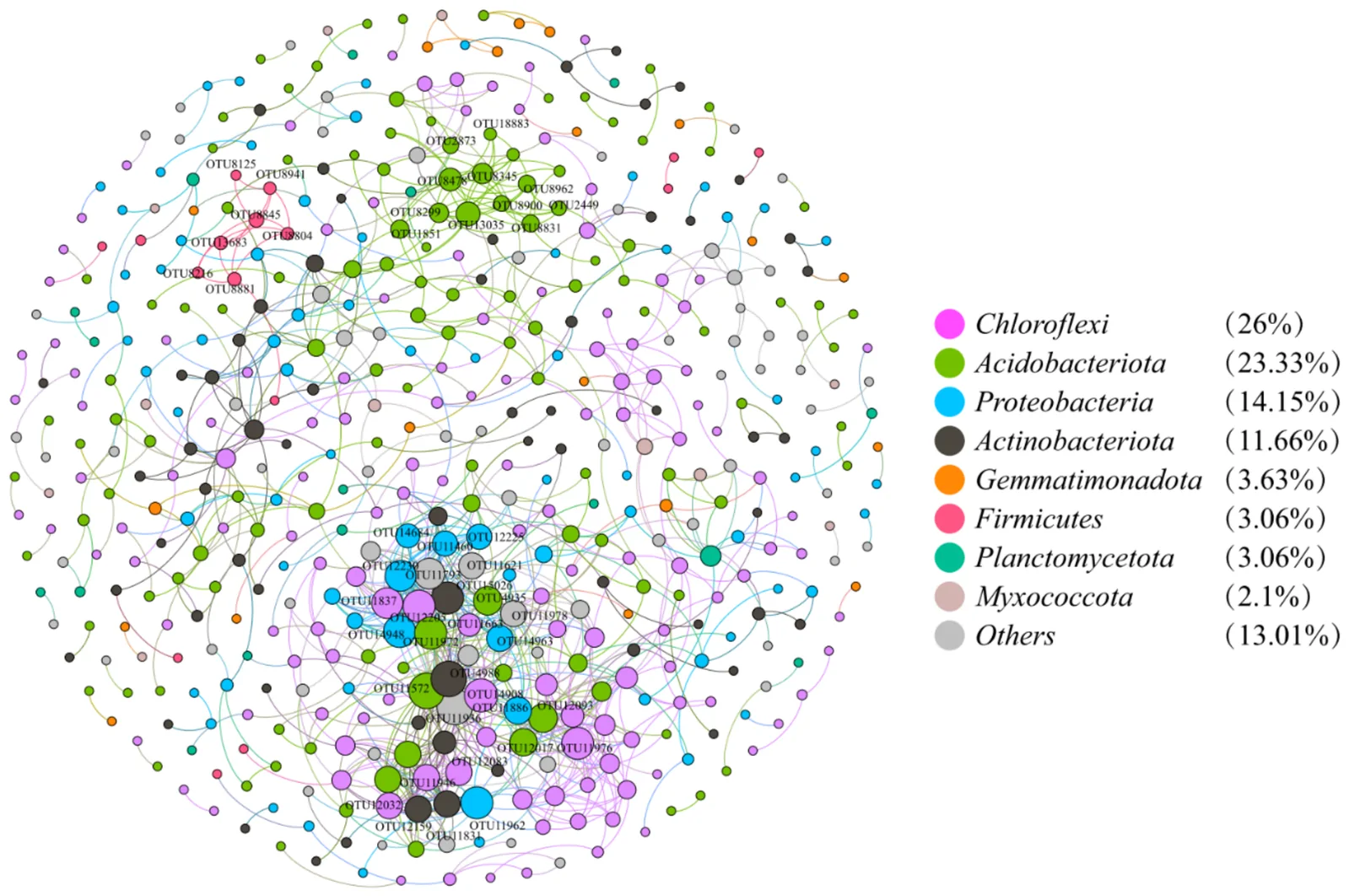
Co-occurrence network diagram of operational taxonomic units (OTUs) with functional groups in soil samples based on pairwise Spearman correlation. The size of each node and the thickness of the lines between two individual nodes are positively correlated with the number of connections.

**Figure 5:**
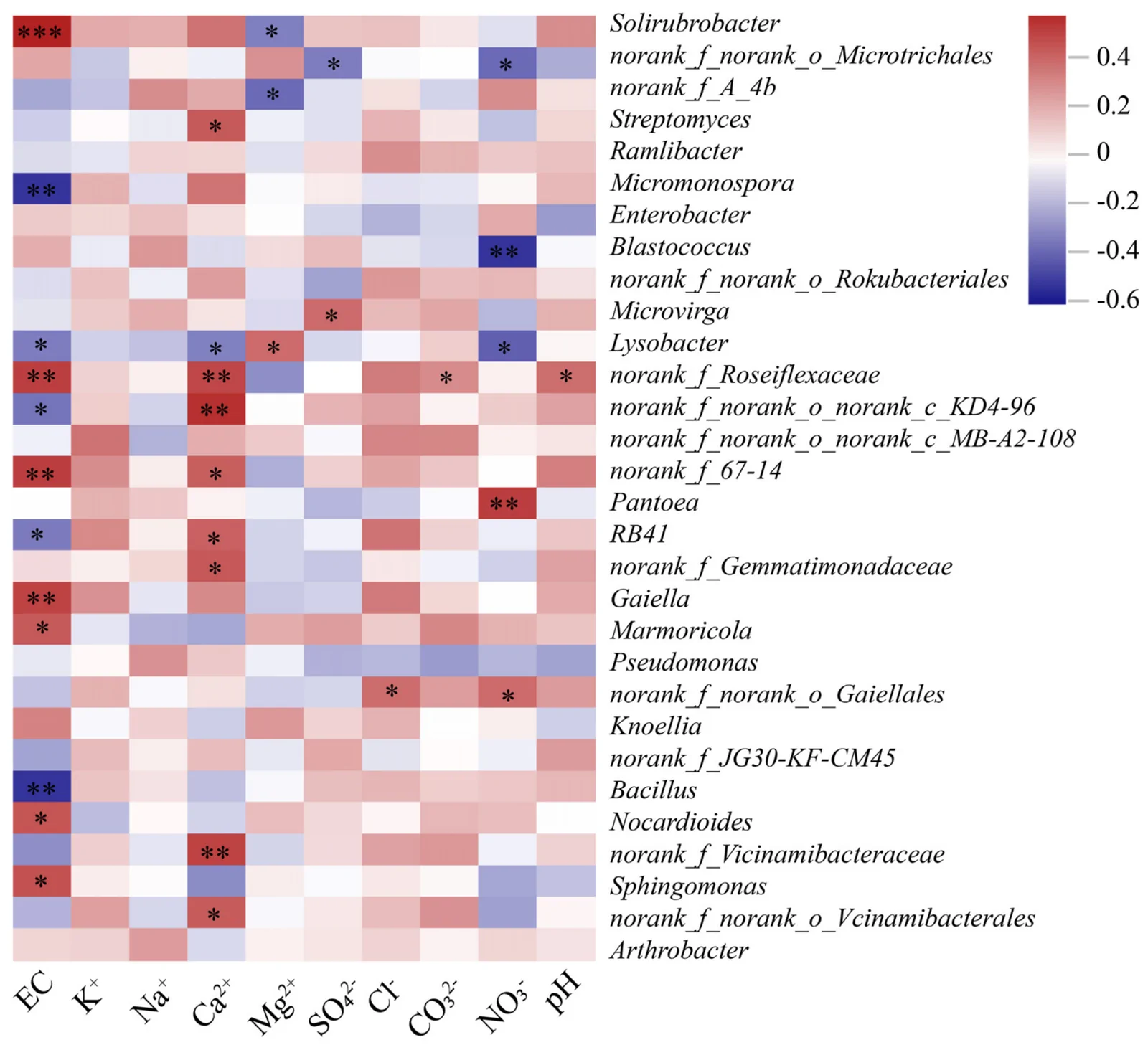
Spearman correlation heatmap showing the associations between soil microbial communities and soil ion parameters. Notes: * *p* < 0.05, ** *p* < 0.01, *** *p* < 0.001.

**Figure 6:**
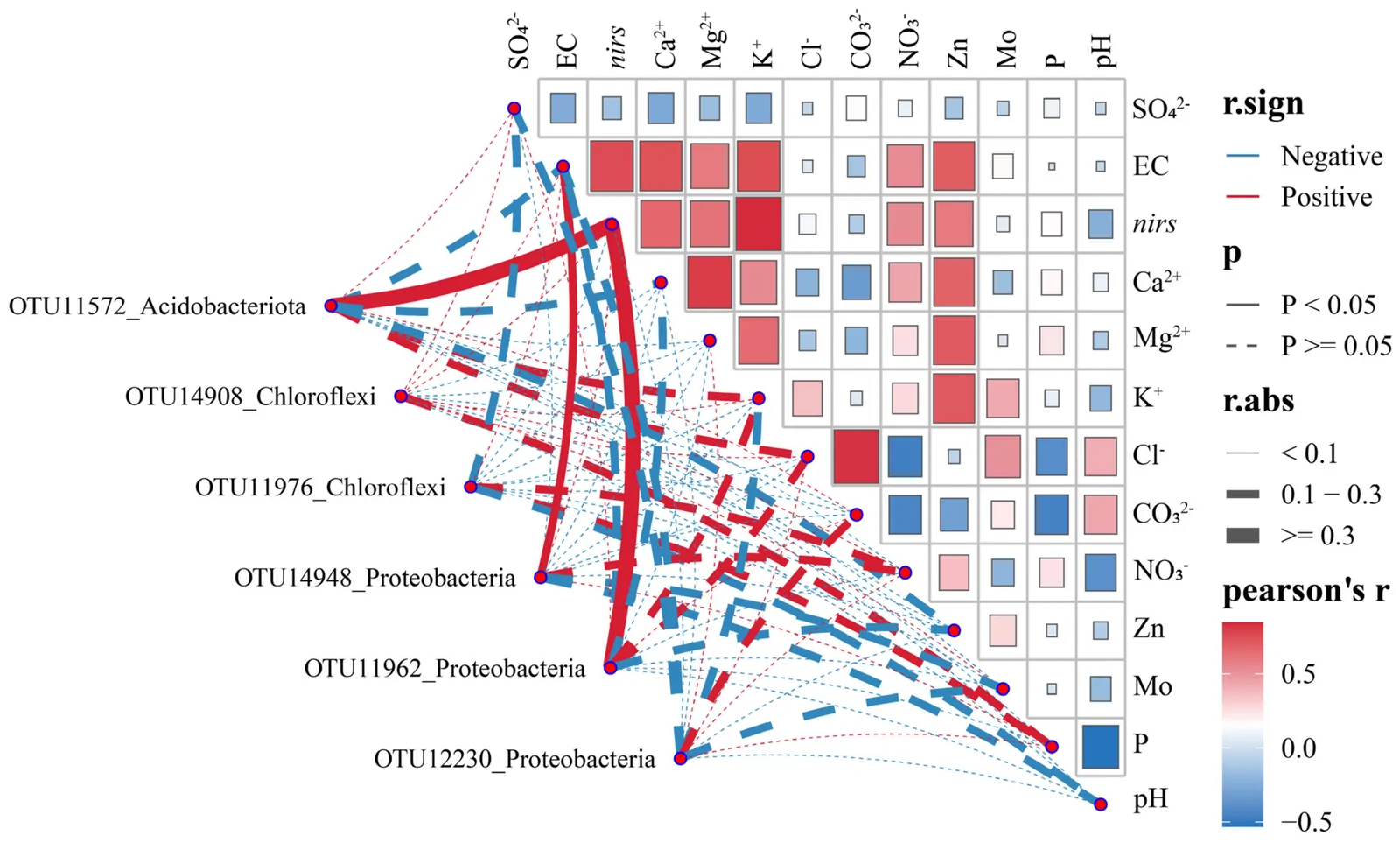
Correlation matrix illustrating relationships between six keystone microbial OUTs and soil physicochemical variables in this study. The plot shows Pearson’s correlation coefficients as color-coded squares, with significant Mantel test correlations marked by curved lines. Line thickness indicates Mantel’s r strength, and color shows the significance of Mantel’s *p* values.

**Table 1:** Key species information in *K*-strategy microbial communities.

OTU	Phylum	Class	Family	Genus	Degree
OTU11572	Acidobacteriota	Acidobacteriae	Subgroup	*Granulicella*	24
OTU14908	Chloroflexi	Ktedonobacteria	Ktedonobacterales	*FCPS473*	22
OTU11976	Chloroflexi	Ktedonobacteria	Ktedonobacterales	*JG30a-KF-32*	21
OTU14948	Proteobacteria	Alphaproteobacteria	Beijerinckiaceae	1174-901-12	21
OTU11962	Proteobacteria	Alphaproteobacteria	Elsterales	*Elsterales*	21
OTU12230	Proteobacteria	Alphaproteobacteria	Beijerinckiaceae	1174-901-12	21

**Table 2:** Standard curves and correlation coefficients of functional genes.

Functional Gene	Amplification Efficiency	Standard Curves	*R* ^2^
*nirS*	100.7%	y= −3.495x + 41.914	0.9977
*nirK*	100.8%	y= −3.12x + 37.748	0.9980
*drsAB*	100.7%	y= −3.373x + 39.225	0.9992

**Table 3:** Simple effects and significance of factors in soil.

Factor	Community	Explains (%)	*Pseudo-F*	*p*
Cl^−^	a	2.5	0.7	0.536
	b	9.2	1.3	0.288
SO_4_^2−^	a	7.5	2.5	0.090
	b	5.5	0.8	0.336
Mg^2+^	a	16.6	3.8	0.010
	b	20.4	4	0.044
Ca^2+^	a	31.5	6	0.004
	b	9.4	1	0.338
NO_3_^−^	a	8.2	4.1	0.074
	b	11.7	1.7	0.164

Note: a, the common bacterial communities among different sampling points; b, the bacterial communities unique to different sampling points.

## Data Availability

All sequencing data were deposited in the NCBI Sequence Read Archive under Bioproject PRJNA586205, with 16S rRNA gene sequencing data available under Biosample numbers SAMN08326675 and raw metagenome data under SAMN08362163. Further inquiries regarding the data can be directed to the corresponding author.
